# Impact of a serious immersive virtual reality game in managing pain during venous or catheter procedures in Pediatric Oncology

**DOI:** 10.31744/einstein_journal/2025AO1327

**Published:** 2025-08-08

**Authors:** Michelle Zampar Silva, Luzia Iara Pfeifer, Barbara Jacomin, Juliana da Costa Feitosa, Raphael de Aro Sousa, José Remo Ferreira Brega, Davi Casale Aragon, Luiz Gonzaga Tone, Carlos Alberto Scrideli, Elvis Terci Valera

**Affiliations:** 1 Department of Pediatrics Faculdade de Medicina de Ribeirão Preto Universidade de São Paulo Ribeirão Preto SP Brazil Department of Pediatrics, Faculdade de Medicina de Ribeirão Preto, Universidade de São Paulo, Ribeirão Preto, SP, Brazil.; 2 Department of Neurosciences and Behavioral Sciences Faculdade de Medicina de Ribeirão Preto Universidade de São Paulo Ribeirão Preto SP Brazil Department of Neurosciences and Behavioral Sciences, Faculdade de Medicina de Ribeirão Preto, Universidade de São Paulo, Ribeirão Preto, SP, Brazil.; 3 Department of Occupational Therapy Universidade Federal de São Carlos São Carlos SP Brazil Department of Occupational Therapy, Universidade Federal de São Carlos, São Carlos, SP, Brazil.; 4 Department of Computing Universidade Estadual Paulista “Júlio de Mesquita Filho” Bauru SP Brazil Department of Computing, Universidade Estadual Paulista “Júlio de Mesquita Filho”, Bauru, SP, Brazil.

**Keywords:** Virtual reality, Neoplasms, Heart rate, Acute pain, Punctures, Catheterization, peripheral, Adolescent, Child

## Abstract

**Objective:**

To analyze the impact of a serious immersive virtual reality game on the physiological and behavioral aspects of pain during peripheral venous access or central catheter puncture in children and adolescents with cancer.

**Methods:**

This was a longitudinal intervention study that included 50 children and adolescents undergoing cancer therapy and peripheral venipuncture or venous catheter puncture (totally implanted or central venous catheter) at two independent time points (control and experimental intervention). The intervention consisted of playing a serious immersive virtual reality game with three-dimensional goggles and a Bluetooth controller. The patients were monitored for alterations in physiological (heart rate and oxygen saturation) and behavioral (agitation, crying, and pain assessed using the Faces Pain Scale-Revised and Visual Analogue Scale) parameters. An agreement analysis was conducted by calculating the weighted kappa coefficient; *p* was obtained using Bowker’s symmetry test.

**Results:**

Use of immersive virtual reality significantly reduced heart rate, improved pain control as assessed by the Faces Pain Scale-Revised, and decreased agitation and crying.

**Conclusion:**

Serious immersive virtual reality games reduce pain and distress during repetitive and uncomfortable procedures such as venipuncture and venous catheter puncture in children and adolescents with cancer. This strategy is simple to implement, cost-effective, and should be considered by health care services assisting pediatric patients with cancer.

## Highlights

■ Immersive virtual reality reduced heart rate, pain, agitation, and crying during pediatric venipuncture.

■ This technology effectively reduced pain during repeated invasive procedures, including catheter punctures.

■ The serious game strategy is low-cost, easy to implement, and well-suited for pediatric oncology care.

## In Brief

Various pain management strategies are employed for children undergoing cancer treatment. Silva et al. prospectively evaluated an innovative approach using an immersive serious game for pain control. The intervention significantly reduced pain perception during venipuncture. Herein, we evaluate immersive virtual reality for pain management in pediatric oncology care.

## Graphical abstract

**Figure:**
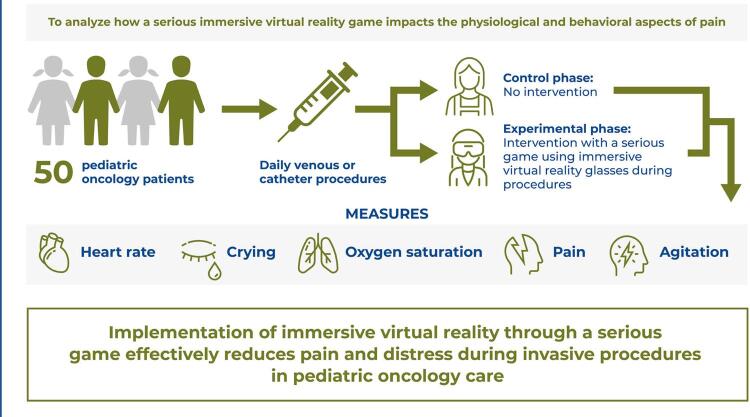

## INTRODUCTION

Pediatric patients undergoing cancer treatment experience multiple symptoms alongside prolonged hospitalization, contributing to negative emotions such as fear, anxiety, and pain. These stressors interfere with the patients’ motor development and ability to engage in daily activities, affecting their quality of life and development.^(1-3)^ The frequent need for invasive procedures causes distress and painful experiences,^(4,5)^ especially during venipunctures for accessing veins and blood collection for laboratory analyses.^(6,7)^ Patients may undergo 25-50 venous catheter punctures during a six-month intensive chemotherapy regimen.^(8)^ Although venipuncture is a routine procedure in hospital units for drug administration and blood collection, the need for repetition can pose a significant challenge to minimizing pain and distress in children.^(9-11)^

Virtual reality (VR) is an interactive technology that provides users with realistic experiences in a synthetic or virtual space.^(12,13)^ Virtual reality can reduce the subjective intensity of pain by modulating multimodal nociception processes.^(14)^ Immersive virtual reality (IVR) involves completely immersing users in a digital environment to provide them with the sensations of a synthetic world. Immersive virtual reality can create pleasant sensations during stressful situations, modify healthcare experiences, and even interfere with them.^(14)^ This can be achieved using technologies such as Head-Mounted Displays (HMDs),^(12)^ which allow three-dimensional (3D) visuals to be projected directly in the patient’s field of vision. This is done via 3D goggles or headsets that simultaneously block all external stimuli. Sensors integrated into the HMD allow the graphical perspective to adapt to the patient’s head movements, creating an immersive experience that is near-authentic. Immersive virtual reality can be combined with serious games, that is, digital games developed for purposes beyond simple entertainment.^(15)^ These games can be used to educate patients on health topics or help them cope with specific medical conditions.

Virtual reality and IVR application during hospital procedures has emerged as a promising research field, with costs decreasing over time and adverse effects being rare.^(16)^ However, the therapeutic application of IVR combined with serious games specifically targeted towards pain management in Pediatric Oncology remains underexplored.

## OBJECTIVE

This study aimed to analyze the impact of serious immersive virtual reality games on the physiological and behavioral variables related to pain during medical procedures (peripheral venous access or central catheter puncture) in patients with pediatric cancer.

## METHODS

### Patient selection

This was a longitudinal intervention study that aimed to compare control (without IVR) and experimental (with IVR) interventions, hereafter designated as “control intervention” and “IVR intervention”, respectively. The analysis was conducted by adjusting to the Bayesian Linear Mixed-Effects Model. The study protocol was approved by the Research Ethics Committee of the *Hospital das Clínicas* of *Faculdade de Medicina de Ribeirão Preto, Universidade de São Paulo* (HC-FMRP-USP), (CAAE: 52862115.4.0000.5440; # 1.411.976).

The study cohort comprised consecutive patients (aged 5-17 years) undergoing treatment at the Pediatric Oncology service of HC-FMRP-USP who required either peripheral venous access or central catheter puncture (totally implanted or central venous catheter) between August 2019 and August 2020. The respective caregivers were also included. The exclusion criteria were: (i) inability to communicate effectively; (ii) severe physical, sensory, or cognitive limitations, as defined by the patient’s health team.

### Data collection

Data were collected by the same health professional from the clinical wards and day hospital sectors of *HC-Criança* (Children’s Hospital of HC-FMRP-USP). The interventions were performed on distinct days and the patients served as their own controls. For the control intervention, data were collected during a routine invasive procedure (peripheral venipuncture or puncture of a totally implanted long-term central venous catheter) and the institution’s normal collection protocol was followed. For the IVR intervention, data were collected during the same invasive procedure within the same setting, but with the incorporation of VR equipment and the serious game *Kimotopia*. Oxygen saturation and heart rate (HR) were monitored during both interventions-before the invasive procedure, during puncture, and 1 min after the procedure. The patients were informed about the required invasive procedures beforehand.^(17)^

### Immersive virtual reality equipment and game design

Equipment for the serious IVR game included adjustable 3D goggles with embedded earphones to block background noise, a light smartphone with a gyroscope (Motorola Z3Play), a Bluetooth controller (Goal Pro VR Z4), a wireless HMD, and a tablet (Samsung) with wireless internet connectivity. All equipment were cleaned with 70% ethyl alcohol before and after use.

**Figure 1:**
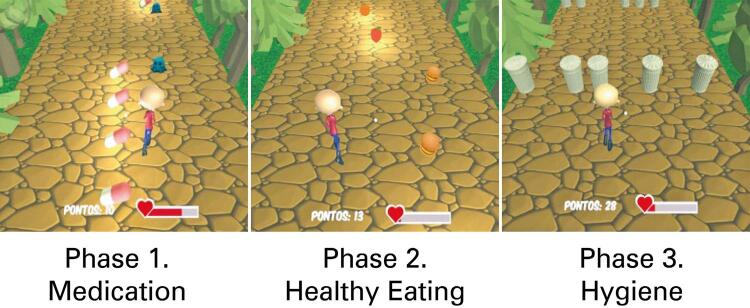
Phases of the game representing the various aspects of oncological treatment

Source: Serious game *Kimotopia*.

The game *Kimotopia* was developed through a collaboration between the *Universidade Estadual Júlio de Mesquita Filho* (UNESP), *Instituto Federal de Educação, Ciência e Tecnologia de São Paulo* (IFSP), and *Universidade de São Paulo* (USP). It incorporates advanced VR technology and simultaneously transmits information. The game addresses health issues related to pediatric patients with cancer^(18,19)^ in an interactive manner, focusing on the three core phases of cancer treatment: medication, healthy eating, and personal hygiene. The tasks are aimed towards educating patients on health maintenance, while scoring them on the completion of each task.^(18,19)^Figure 1 shows representative screen captures of the three phases of the game.

**Figure 2:**
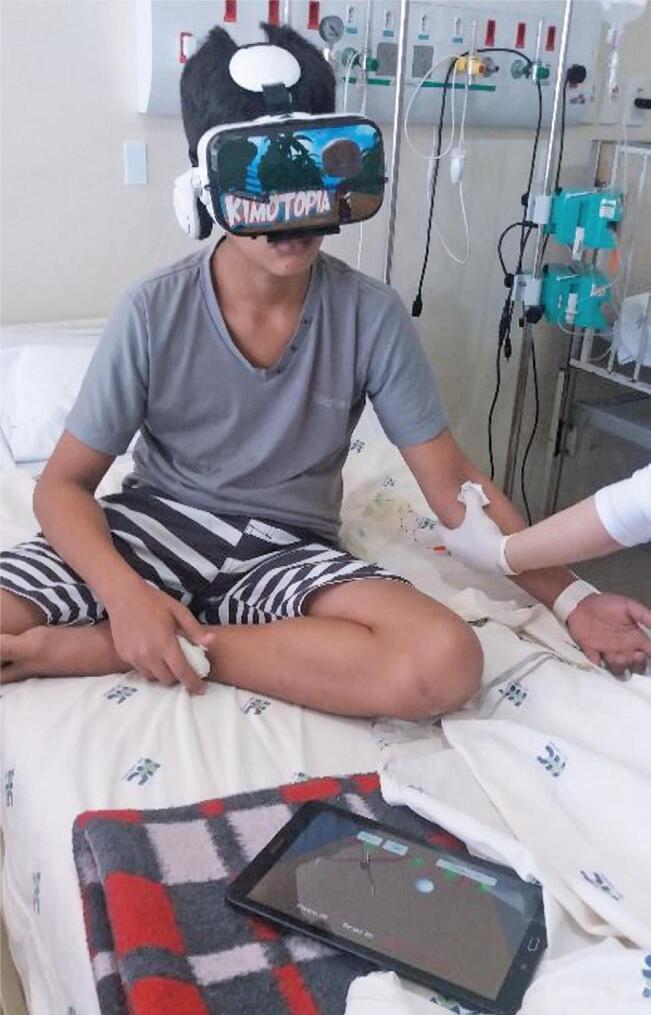
A patient playing the game *Kimotopia* during peripheral venipuncture

The ability to control the game using head movements enables active patient participation with minimal body movement. This makes the equipment suitable in situations where the hands are restricted (such as during peripheral venous access or central catheter puncture). Figure 2 shows the game played by a patient during venous puncture.

### Assessed variables

Visual Analogue Scale (VAS): This tool assesses acute procedural pain perceived by patients. It consists of a straight horizontal line limited to 100mm in length, which represents the continuum of the painful experience. It has the words “no pain” on the left and “worst possible pain” on the right as the extreme limits of pain.^(20)^

Faces Pain Scale-Revised (FPS-R): This tool assesses the intensity of self-reported pain. It comprises a series of six neutral facial expressions (without tears or smiles) corresponding to a 0-10 metric of increasing pain.^(21)^

Face, Legs, Activity, Cry, and Consolability-Revised (FLACCr): The FLACC scale was adapted to evaluate two scale domains: crying and agitation.^(22)^ The scores varied from 0 to 2 for each domain.

Behavioral and physiological measurements: a pulse oximeter was used to monitor patients for physiological alterations (HR and oxygen saturation). Measurements were taken before the invasive procedure, at the time of puncture, and 1 min after completion of the invasive procedure.

### Statistical analysis

The differences between the means obtained for each intervention and their respective 95% confidence intervals (95%CI) were calculated. The data were adjusted using regression analysis. Univariate and multivariate analyses were performed using a Bayesian approach.^(23)^ The pain covariables were: number of punctures, whether the same nurse performed the invasive procedure in both interventions, crying, and agitation. To verify the agreement between the control and IVR data, the weighted kappa coefficient was calculated together with 95%CI. The interpretation of kappa coefficient follows Landis et al.’s^(24)^ criteria: <0 (bad), 0.01-0.20 (weak), 0.21-0.40 (regular), 0.41-0.60 (moderate), 0.61-0.80 (strong), and 0.81-1.00 (almost perfect). Bowker’s symmetry test was employed to determine p. Low p indicate a significant difference between the interventions regarding pain scales, crying, and agitation.^(24)^ For the analysis, SAS (version 9.4) and R (version 4.0.2) software were used.

## RESULTS

Sample size was calculated based on the annual cohort of 70 new pediatric cancer cases at the pediatric oncology sector of HC-FMRP-USP. With 90% power to detect interventional differences and an assumed standard deviation of 4.5, a sample size of 48 patients was deemed sufficient to assess the effect of IVR intervention on pain during peripheral venous access or central catheter puncture.

### Patient characteristics

This study included 77 children and adolescents. Twenty-seven patients were excluded at follow-up for the following reasons: 13 were not required to return to the ward/outpatient clinic; 4 quit the study during the IVR intervention; 4 progressed with neurological problems that prevented them from being assessed during the IVR intervention; 3 participated only in the pilot study; 1 was older than 18 years; 1 underwent a different invasive procedures between the control and IVR interventions; and 1 died prior to IVR intervention. Thus, the final sample consisted of 50 patients, where 74% (37) and 26% (13) underwent peripheral venipuncture and puncture of the totally implanted-long-term central venous catheter (LTCVC), respectively. The median time between the control and IVR interventions was 5 days (1^st^ quartile 25%: 2 days; 3^rd^ quartile 75%: 21 days). For 30 patients, both interventions occurred during the same hospitalization period, whereas for the remaining 20, the interventions occurred during distinct hospitalizations.

Among the participants, 28 (56%) were male. The mean age was 10.44 years (+3.79 years), with 25 aged <12 years and 25 aged 12-17 years. The mean hospital stay for the pediatric ward patients was 12 days. The patients were diagnosed with leukemia (24%), lymphoma (24%), Central Nervous System (CNS) tumors (16%), hematological diseases (16%), bone tumors (10%), abdominal tumors (6%), or malignant neoplasms of the orbit and histiocytosis (4%). The reasons for hospitalization at the time of the control intervention were as follows: 27 patients were undergoing chemotherapy, 7 had an oncological diagnosis, 8 had neutropenic fever, 3 were undergoing preoperative assessment, 2 were postsurgical patients, 3 were being prepared for bone marrow transplantation, and 1 was undergoing radiation therapy. The reasons for hospitalization at the time of the IVR intervention were as follows: 26 patients were undergoing chemotherapy, 7 had an oncological diagnosis, 9 had neutropenic fever, 2 were undergoing preoperative assessment, 4 were postsurgical patients, and 3 were being prepared for bone marrow transplantation.

At the pediatric ward of *HC Criança*, routine peripheral venipunctures for blood collection lasted 4’12’’ (control intervention) and 3’13” (IVR intervention) on average. For totally implanted-LT-CVC puncture conducted at the chemotherapy center of the hospital, the routine procedure lasted 2’23’’ (control intervention) and 1’34’’ (IVR intervention) on average. During the interventions, time measurement commenced when the professional first handled a procedural instrument after all instruments had been appropriately positioned.

### Pain assessment

With regard to the results obtained by applying the FPS-R after the control intervention, 25 patients reported weak pain (0-2), whereas 13 reported intense pain (8-10). After the IVR intervention, the number of patients with weak pain increased to 36, whereas the number of patients with intense pain decreased to 6. Weighted kappa (95%CI) was 0.39 (0.16; 0.61), and Bowker’s symmetry test gave a p-value 0.02 which showed a significant difference (p<0.05) and regular agreement (weighted kappa, 0.21-0.40) between the control and IVR interventions.

During VAS application, 27 patients reported weak pain intensity (0-29 mm on the scale) during the control intervention, while 9 patients reported highly intense pain (80-100 mm on the scale). After the IVR intervention, 37 patients reported experiencing weak pain, while only 4 experienced intense pain (weighted kappa (95%CI)=0.30 (0.07; 0.53); Bowker’s symmetry test: p=0.08). Thus, VAS analysis did not identify a significant difference between the control and IVR interventions.

### Assessment of physiological variables related to pain

For the crying domain, the FLACCr assessment assigned scores of 0 for 29 and 39 patients during the control and IVR interventions, respectively. Eleven patients cried, screamed, sobbed, or frequently complained during the control intervention, whereas only two patients displayed such behaviors during the IVR intervention. Weighted kappa (95%CI) of 0.34 (0.13; 0.55) and Bowker’s symmetry test (p<0.0001) showed significant differences between the interventions.

For the agitation domain, 25 remained sitting at puncture initiation, while 41 patients lay down in silence, showing less agitation. In contrast, 12 patients displayed shaking and tremors, while 2 exhibited rapid breathing, indicating extreme agitation. Thus, the Control and IVR intervention groups showed significant differences (p<0.05) with regular agreement (weighted kappa, 0.21-0.40).

**Figure 3:**
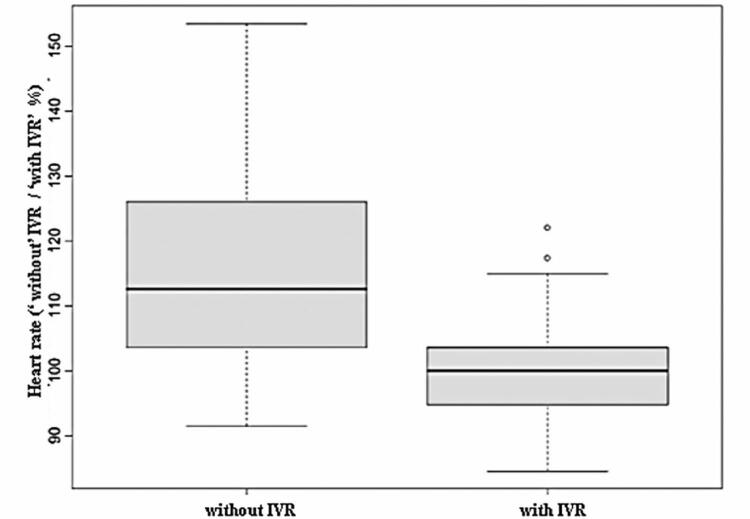
Heart rate analysis during the control and immersive virtual reality interventions

**Figure 4:**
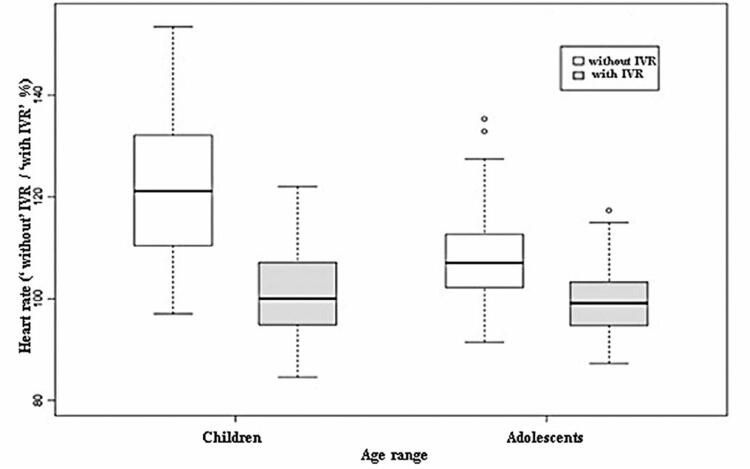
Heart rate analysis of the control and immersive virtual reality intervention subgroups (children and adolescents)

The mean HR decreased during the IVR intervention compared to that during the control (p<0.05) (Figure 3). Two analysis models were used: a univariate model (direct comparison between the interventions) and a multivariate model (comparison between the interventions considering the covariable number of punctures, whether the same nurse performed the invasive procedure in both interventions, crying, and agitation). Table 1 lists the 95%CI, defined as the differences between the mean HRs of the control and IVR interventions. Both the univariate and multivariate analysis models showed that HR reduced significantly during the IVR intervention, even when subgroups of adolescents and children aged <12 years were analyzed (Table 2 and Figure 4).

**Table 1:** Differences in mean heart rate between control and immersive virtual reality intervention groups with 95%CI, based on regression analysis models

Regression analysis model	Variable	Difference between the mean	95%CI
Univariate	HR	-15.9	(-20.54; -11.25)
Multivariate	HR	-10.6	(-16.67; -4.53)

95% confidence intervals that do not include zero indicate significant differences.

**Table 2:** Differences in mean heart rate between control and immersive virtual reality intervention subgroups (children and adolescents), with 95%CI based on regression analysis models

Regression analysis model	Comparison	Difference between the mean HR	95% CI
Univariate	Children (control–IVR interventions)	22.79	(16.78;28.81)
	Adolescents (control–IVR interventions)	9.00	(2.99;15.01)
Multivariate	Children (control–IVR interventions)	18.43	(10.34;26.52)
	Adolescents (control–IVR interventions)	7.38	(0.70;14.06)

95% confidence intervals that do not include zero indicate significant differences.

In terms of oxygen saturation, the univariate analysis model provided a difference of 0.04 between the mean oxygen saturation of the interventions, while the 95%CI was -0.48-0.40. The multivariate analysis model showed a mean difference of -0.07 between the interventions, with a 95%CI= -0.68-0.54. Since zero lies within the range for both models, the differences between the interventions were not considered significant.

## DISCUSSION

Adequate pain management, an indicator of quality of life and healthcare^(4,25)^ must address the physical, psychosocial, and spiritual aspects of both patients and their respective families.^(26)^ Since pain is inherently subjective, it is characterized as a highly complex and multidimensional symptom. The patient’s self-report is the gold standard for determining the presence of a symptom,^(27,28)^ and the International Association for the Study of Pain clearly states that an individual’s report of pain must always be considered. Multiple instruments are often necessary to obtain a reliable picture of the pain experienced by children and adolescents. Although children have little life experience, they can provide reliable self-reports to enhance the accuracy of their symptom assessment.^(2,29)^

In this study, the patients reported fear of invasive procedures. Studies have found that needle-related discomfort is high among children with cancer^(6)^ and that fear may impact the efficacy of distraction-based interventions.^(30,31)^ Although anxiety was not assessed in the current study, negative memories of a procedure may result in exaggerated responses to pain, thereby raising anxiety during subsequent procedures.^(32)^ Notably, IVR intervention has been proven to be effective in reducing pain related to needles and distress.^(30)^

The use of IVR to control pain in children is increasingly being investigated. A recent study^(33)^ on the use of IVR during blood collection for laboratory analysis observed a less negative emotional appearance, less pain, and lower fear scores compared to those of the Control Group. Based on the FPS-R scale, patients experienced reduced pain when IVR was employed during venipuncture or puncture of a totally implanted-LT-CVC. Many authors consider this scale to be adequate for children and adolescents as it effectively reflects pain-related discomfort.^(34)^

Venipuncture and puncture of a totally implanted-LT-CVC require the patient to maintain a stable position, keeping the limbs as immobile as possible during the procedure.^(35)^ In this sense, choosing adequate IVR equipment for use in different pediatric clinical settings is essential. The choice of IVR, aided by an HMD, 3D goggles, and head movement with limited hand use, provided the health team with free access to the upper limb or the central catheter in the thoracic wall.

Analysis of pain using physiological assessments is extremely reliable.^(36,37)^ The benefits of VR in clinical situations have been shown to reduce affliction in children using distraction-based VR strategies.^(38)^ In such scenarios, the positive outcomes are evident from patients’ reduced physiological excitation, pain scores, HR, and anxiety sensitivity indices. Heart rate and anxiety sensitivity indexes, evaluated here, supported the positive effects of using a serious IVR game. Behavioral and cardiac indicators have been suggested as unique aspects of the nociceptive response during vaccine administration in healthy children.^(39)^ A VR intervention study found that children in the VR intervention group had significantly lower HR than those in the Control Group.^(40)^ Aligned with this observation, another study found that children who used VR did not experience as much pain and anxiety as those in the Control Group.^(36)^ A systematic review of 15 studies compiled by Gautama et al.^(41)^ involving adult and pediatric cancer patients and the use of IVR showed that IVR significantly reduces anxiety, depression, fatigue, and systolic blood pressure. In pediatric patients undergoing chemotherapy, IVR significantly alleviated pain and anxiety but not HR, which is in contrast with the results of the present study.

Systolic and diastolic blood pressure, respiratory rate, oxygen saturation, and HR were measured to assess other physiological measures of pain.^(42)^ Here, although we analyzed oxygen saturation, we did not find any significant differences between the interventions with respect to this variable.

Despite the role of distraction in pain during invasive procedures, to date, no study has adequately explored the possibility of using a serious IVR game to control pain during peripheral venous access or central catheter puncture in children with cancer. In this study, we followed determinations^(43)^ that suggested engaging a multi-professional team familiar with the target population to develop a serious game addressing a clearly identified need. The phases created for the game were based on the demands of pediatric cancer patients at our institution.

Previous studies have reported the adverse symptoms of nausea and dizziness in patients undergoing VR interventions during needle-related procedures.^(44)^ Such symptoms were not observed in this study. We believe that incorporating patient feedback in the study design assured a sense of safety and acceptance of the IVR intervention.

The use of VR in pediatric cancer care has extended beyond pain management during chemotherapy. A recent German study demonstrated the feasibility of using VR via smartphone apps to prepare children for proton therapy sessions, with promising initial results.^(45)^ In our study, implementing the IVR resource proved viable for numerous reasons: the equipment was portable and completely wireless; therefore, it could be easily carried by a health professional and connected at different clinical settings; the professional that monitored the equipment did not require any specific qualification; the estimated cost of the IVR equipment was low (approximately 500 dollars); and the equipment was durable.

### Limitations of the study

This study has some limitations. First, patients had to be excluded for various reasons. For instance, data were collected during the COVID-19 pandemic, which did not allow 13 patients to be rehospitalized for IVR intervention. Second, given that the study involved routine peripheral venous access or central catheter puncture, some covariables may not have been adequately controlled. Third, although implementing the IVR intervention was beneficial in terms of reducing pain during invasive procedures, the procedures required at least two staff members—one nurse performing the puncture and one professional handling the electronic equipment.

## CONCLUSION

A serious immersive virtual reality game can effectively reduce the pain experienced during inherently painful procedures, such as peripheral venous access or central catheter punctures, as demonstrated by both physiological and behavioral parameters. Future research must analyze the experience and aspects of immersive virtual reality intervention, combining new self-reports with other physiological indicators of pain such as anxiety, fear, and stress. Similar interventions can be adopted during other prolonged medical interventions.
